# Intelligent Nanomaterials for Wearable and Stretchable Strain Sensor Applications: The Science behind Diverse Mechanisms, Fabrication Methods, and Real-Time Healthcare

**DOI:** 10.3390/polym14112219

**Published:** 2022-05-30

**Authors:** Veluru Jagadeesh Babu, Merum Anusha, Merum Sireesha, Subramanian Sundarrajan, Syed Sulthan Alaudeen Abdul Haroon Rashid, A. Senthil Kumar, Seeram Ramakrishna

**Affiliations:** 1 NUS Centre for Nanotechnology and Sustainability, Department of Mechanical Engineering, National University of Singapore, Singapore 117581, Singapore; vsiri09@gmail.com (M.S.); sulthanshine@outlook.com (S.S.A.A.H.R.); seeram@nus.edu.sg (S.R.); 2 Department of Pharmacology, S V Medical College, Dr NTR University of Health Sciences, Vijayawada 517501, India; dr.anusha.m@gmail.com; 3 Centre for Advanced Materials and Industrial Chemistry (CAMIC), School of Science, RMIT University, Melbourne, VIC 3001, Australia; 4 Advanced Manufacturing Laboratory, Department of Mechanical Engineering, National University of Singapore, Singapore 117581, Singapore; mpeask@nus.edu.sg

**Keywords:** wearable and stretchable strain sensor, intelligent nanomaterials, human motion monitoring, mechanism, fabrication techniques

## Abstract

It has become a scientific obligation to unveil the underlying mechanisms and the fabrication methods behind wearable/stretchable strain sensors based on intelligent nanomaterials in order to explore their possible potential in the field of biomedical and healthcare applications. This report is based on an extensive literature survey of fabrication of stretchable strain sensors (SSS) based on nanomaterials in the fields of healthcare, sports, and entertainment. Although the evolution of wearable strain sensors (WSS) is rapidly progressing, it is still at a prototype phase and various challenges need to be addressed in the future in special regard to their fabrication protocols. The biocalamity of COVID-19 has brought a drastic change in humans’ lifestyles and has negatively affected nations in all capacities. Social distancing has become a mandatory rule to practice in common places where humans interact with each other as a basic need. As social distancing cannot be ruled out as a measure to stop the spread of COVID-19 virus, wearable sensors could play a significant role in technologically impacting people’s consciousness. This review article meticulously describes the role of wearable and strain sensors in achieving such objectives.

## 1. Introduction

**Figure 1:**
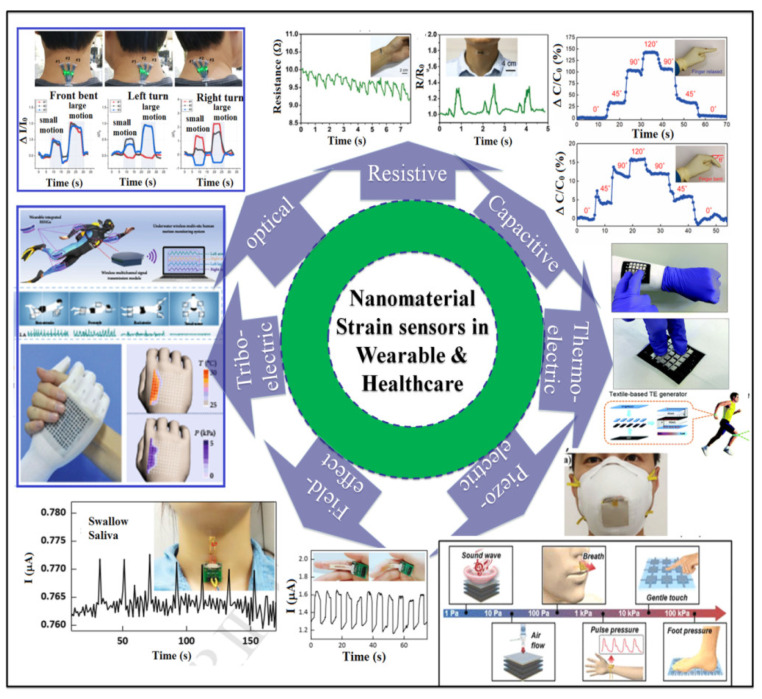
Nanomaterials-based strain sensors for wearable and healthcare applications.

In recent decades, nanostructured materials such as nanowires (NWs) [1,2,3,4], nanoparticles (NPs) [5,6,7], nanofibers (NFs) [8,9,10,11], and nanomaterials such as carbon nanotubes (CNTs) [12,13,14,15], carbon fibers (CFs) [16,17], graphite [18,19,20,21], and graphene (GE) [22,23] have earned a wide focus in research due to their eminent physical, mechanical, chemical, and electrical properties. In addition, the ease in cost, synthesis of these nanostructures in different morphologies, and further fabrication of devices for an extensive range of applications have made them essential in the field of device fabrication [24,25]. In general, when a nanostructured material is stimulated with external factors such as temperature, bias, and strain/pressure, it leads to a change in electrical resistance/current or voltage [26,27]. The change in energy from one form to another facilitates its use as a sensor for human motion detection and also the possibility of it acting as an electronic skin [28] for applications such as medical diagnosis [29,30,31] and wearable electronics [32,33,34]. The working mechanisms behind the wearable and strain sensors can be classified as resistive [27,35,36,37,38,39,40], capacitive [15,41,42,43,44,45], thermoelectric [46,47], piezoelectric (PE) [48,49,50,51,52], triboelectric [53,54,55,56,57], field-effect [32,58,59], and optical [60,61]. The sensing materials and sensitive layer play a vital role in the above operating principles, which in turn dictate the choice of application. To achieve sensor performances, such as sensitivity, selectivity, and stability, various types of sensitive materials have been considered for sensor fabrication in the past, including organic, carbon, metal, semiconductor, and hybrid materials. However, the stretchable nature of the materials can be significantly improved by choosing intrinsic stretchable materials such as ionic conductive hydrogels as the sensing layer. The major challenge in the current field of research is to fabricate a strain sensor with high sensitivity and stretchability. Chen et al. fabricated a highly sensitive and stretchable strain sensor, based on graphene nanosheets (a combination of planar graphene and crumpled graphene) with a gauge factor of 20.1 and a strain performance of 105%, which effectively monitored sensitivity as well as stretchability at the same time [62]. In another study, the authors of [63] fabricated a highly sensitive and stretchable strain sensor based on Ag NPs and Ag NWs into an aid filler of polydimethylsiloxane (PDMS) to enhance the sensitivity of the sensing layer. Ag NPs or Ag NWs with PDMS showed a very highly tunable gauge factor up to 3766 that enabled the sensors to be uniquely high-performance strain sensors that accurately detected physical movements such as talking, bending of a finger, wrist raising, and walking [63]. A recent study demonstrated using In_2_O_3_ Nanoribbons (NRs) and NWs as Field-Effect Transistor (FET)-based strain sensors with gold as gate electrodes used for glucose detection in body fluids such as sweat and saliva [64]. These are very few examples of such uses within the extensive literature available. This report is a scientific collection of the existing wearable/SSS deploying nanostructures into stretchable features such as sensing layers, sensor substrates, and other forms of requirements used in the fabrication. A detailed demonstration of challenges in fabricating wearables/SSSs are also emphasized. Figure 1 gives an overview of various modes of operation of wearables/SSSs.

## 2. Nature vs. Technology

**Figure 2:**
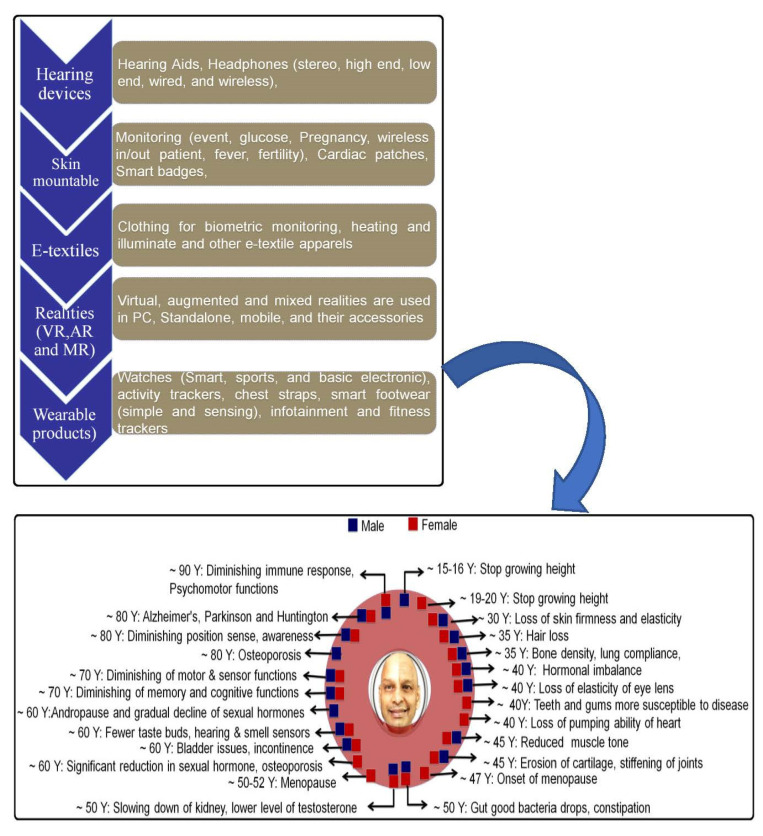
Nature vs. technology.

Aging is inevitable to human life; hence, technological support is an artificial tool to serve the shortcoming of aging (presented on the right side of Figure 2). The authors acknowledge that there is no replacement to nature, but wearable and strain sensors can be a mild alternative to detect different levels of serviceability of the human body. Professor Seeram Ramakrishna from the National University of Singapore uses photographs to depict the phenomena of natural aging subject to genetics, lifestyle, and other environmental factors.

### 2.1. Components of SSS

This section identifies the different types of wearables/SSSs and their different modes of operating principles. As presented in Figure 1 and Figure 2, most strain sensors are based on nanomaterials of different kinds and are extensively used in wearable applications such as electronic skin or skin-mountable sensing applications [65,66,67,68,69]. Whereas the nanomaterials are responsible for the sensing part, other components such as substrates, electrodes, and dielectric materials also play a vital role in fabricating an efficient strain sensor. The below sections explain the roles, materials, and importance of the sensor’s fabrication components such as supporting material, sensitive materials, and electrode materials in detail.

### 2.2. Substrates

In general, PDMS polymers are used as a substrate for many electronic devices due to their high sensitivity and low detection ranges for various applications [70], but they are vulnerable to environmental factors, making them less practical for real-time applications. Therefore, a variety of substrate materials are used for SSS, including polyurethane sponge shapes [71,72,73,74], poly (ethylene terephthalate) (PET) [75], polyimide substrate [76], tissue paper [77], textiles [78], elastomeric fibers [79], and cotton [80]. Core–shell structured Polyurethane/cotton/CNTs have been reported, a substrate with a high stretchability of 300%, and this substrate is stable for up to 300,000 cycles [81]. Silk-molded nanostructured substrates have been used as strain sensors for real-time monitoring, exhibiting a fast response, high stability, and high sensitivity [78]. Graphene nanoflakes show a high sensitivity in flexible sensors [82]. In addition, rubbers [83,84,85,86,87,88,89,90,91,92,93], thermoplastic polymers, [94,95,96,97,98,99,100,101,102,103,104,105,106,107], medical adhesive films [108,109,110], and natural fiber-based materials such as cotton, wool, and flax have also been widely used as supporting substrates for the fabrication of SSS [111,112,113,114,115,116,117,118]. Thermoplastic polyurethane (TPU) [68], polystyrene-based elastomers [69], and PDMS [119] are some of the materials employed in stretchable optical strain sensors (OSS) substrates.

### 2.3. Sensitive Materials

As mentioned earlier, flexible SSSs use different types of nanomaterials and are categorized as organic sensing materials and inorganic sensing materials. Organic sensing materials such as CNTs [71,74,120], graphene [121,122], conductive polymer NFs [123], carbon black [124,125] and graphene composite with inorganic materials [126] are widely used. Ryu et al. fabricated elastic strain sensors with highly oriented CNTs grown on a flexible substrate in which the device can stretch more than 900% and the gauge factor is about 64 [127]. Park et al. reported PE flexible sensors with PVDF-TrFE electrospun NFs performing ultrahigh stability [123]. Other materials such as PEDOT: PSS [128], polyaniline (PA) [129,130,131], and hybridized graphitic N-doped silk NFs are promising for SSS [132]. Electrospun nanofibrous membranes possess tunable porosity, surface-area-to-volume ratio and ultra-lightweight properties, which make them a perfect material for flexible strain sensors. PA/PVDF composite fibers were generated using electrospinning and have been used to fabricate SSS, which efficiently had a stretchability higher than 110%. Polypyrrole (PPy) is another conductive polymer composite with CNTs and has been used to fabricate flexible sensors [133]. The sensors made of PPy and silver aero sponge flexible sensors demonstrate a low detection of about 4.93 Pa [134] and are widely used as sensitive materials. Some active sensing materials are inorganic materials, which show high sensitivity, low cost, and ease of fabrication methods. However, inorganic materials are not directly used for monitoring human motion since they are rigid. To improve flexibility and stretchability, inorganic materials such as ZnO and Ag are composited with polystyrene and produce NWs and are further used to fabricate strain sensors [135,136]. A layer-by-layer method is also reported to fabricate carbon black nanosponges, which can effectively be used to detect tiny pressures of 91Pa at about 0.2% strain and monitoring of large human activities [137].

### 2.4. Electrode Materials

In some cases, active material can also act as an electrode for flexible sensors. In some cases, contact area deformation is also a key factor. Various materials are used as electrode materials. CNT and graphene are widely used as an electrode material since their crystal structure is suitable for high conductivity and thereby makes them as an effective material for flexible sensors [35,71,72,137]. In further sections, a detailed explanation on electrode materials is explained in detail.

## 3. Different Types of Stretchable Strain Sensor

### 3.1. Resistive-Type Strain Sensors

Resistive-type strain sensors are typically composed of active sensing materials combined with flexible and stretchable supporting substrates [138,139]. The sensitive materials can be in the form of conductive micro-/nanomaterials-based polymer composites, thin films, or conductive yarns/fabrics. The conductive network of sensitive materials serves as a resistor under the application of a potential bias (voltage). When stretched, the electrical resistance of the sensitive network changes as a function of the applied mechanical strain. The resistance variations upon stretching originate from the geometrical changes, i.e., length and cross-sectional area [65,66,139]. The sensitive materials recover back to their initial state after experiencing a tensile/compressive strain, which is measured and makes it the principle behind resistive-type sensors.

**Figure 3:**
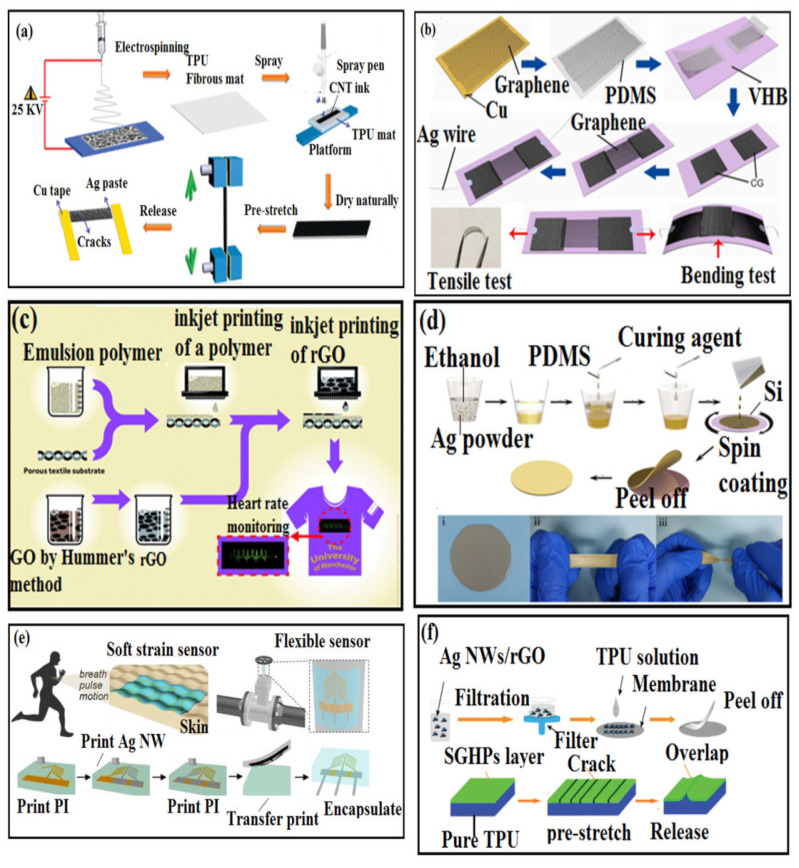
(**a**) Schematic of the fabrication process and crack formation of CNT/TPU composites [146]; (**b**) schematic diagram of transfer printing of graphene to make a strain sensor, tensile test, and strain sensor under bending test [62]; (**c**) inkjet printing of an organic-nanoparticles-based surface, pretreat onto textiles to enable all inkjet-printed graphene e-textiles [141]; (**d**) Ag/PDMS-based sensor fabrication process and an image depicting flexibility under stretching and twisting [147]; (**e**) wearable, skin-conformal soft strain sensor for the monitoring of breathing and pulse and motion and a flexible, soft strain sensor [148]; (**f**) fabrication of Ag NW/RGO/TPU composites and its stretching and relaxation process [149].

Resistive-type WSSs have been fabricated using different synthesis techniques, namely, electrospinning, spray coating, inkjet printing, chemical route synthesis, sputter-coating, printing, liquid phase blending, and filtration. As shown in Figure 3a, a resistive-type SSS with reduced graphene oxide (rGO) furnished with thermoplastic polyurethane (TPU) electrospun nanofibrous strain sensors were attached on skin or clothes to monitor various human motions [94]. When the sensitive material is applied with a stretch of about 0.5% strain, a quick response was obtained in 200 ms, which makes it an exceptional sensor to monitor even minute muscle motion, breathing postures, and pulses [94]. Chen et al. reported that the crumpled graphene/PDMS via a transfer printing method (see Figure 3b) could bear a strain of up to 105% with a gauge factor of about 337.8, which in turn can measure sensitivity and stretchability concurrently for monitoring movements of joints [62]. As one of the most effective physical coating methods, ink-jet printing is widely used for the deposition of graphene (see Figure 3c) [140]. Using an ink-jet printing technique, a polyester/cotton woven fabric was pretreated by spraying a layer of 200 μm thick styrene/divinylbenzene emulsion as a flat substrate. Furthermore, a GO was coated on the flat surface of the fabric by printing, and finally the GO was reduced to rGO by vitamin C. The prepared rGO showed good electrical properties (resistance of 1.18 Ω sq^−1^), which means it can be used for sensitive applications such as heartbeat monitoring [141]. The 3D printing process has been used to fabricate CNTs/GO-based SSS [142]. A simple template method presented in Figure 3d was used to fabricate rGO/deionized water filled in Eco-flex lines, which have highly stretchable strain sensing applications [143].

A composite film made of graphene NPs and PDMS with high conductivity has been reported for WSS [83]. Initially, these graphene NPs were transferred onto the PDMS substrates using medical tape and pressed mechanically to obtain high conductivity [83]. A spray-coating technique was used for CNTs on elastic rubber film, producing highly SSS [84]. In another study, CNTs/carbon black hybrid network spin coated on PDMS substrate for synergistic conductive strain sensors [144] was reported. A conductive fiber made from Ag NWs and polyurethane composites, fabricated wearable sensing devices and the bonding between Ag and polyurethane were also investigated [145] (see Figure 3d). In another study, Ag/polyurethane micro-flakes-based electrodes were made using PDMS as supporting material, NaCl as sacrificial agent, and polyurethane/carbon black as a sensing layer [95].

This novel structure minimized in-plane stretching disturbance, leading to customizable SSS. A chemical vapor deposition was used to grow vertically aligned CNT bundles followed by rolling and transfer onto the Si substrate, which thereby fabricated a stretchable strain sensor [142]. Aerosol jet printing of polyimide (PI)/Ag NWs enables multi-functional strain sensors with tunable resistance; initially, PI and Ag NWs were deposited on a glass slide with spin-coated PMMA (see Figure 3e) [148]. Strain sensors based on multiple-layered structures demonstrate capabilities such as strong adhesion and conformal lamination on different surfaces without the use of conventional fixtures and/or tapes. Chen et al. synthesized a functionalized organic nanoparticle embedded in a hydrophobic breathable coating on textiles. Subsequent inkjet printing of continuous conductive electrical path onto the pretreat coating reduced the sheet resistance of graphene-based printed e-textiles by three orders of magnitude from 1.09 × 106 Ω/sq. to 2.14 × 103 Ω/sq [146,149] (see Figure 3f).

### 3.2. Capacitive-Type Strain Sensors

Wearable capacitive-based strain sensors are often fabricated by sandwiching an insulating film known as a dielectric layer between two stretchable electrodes [66]. Under an applied voltage, the accumulated opposite charges on each electrode cannot flow through the dielectric layer, yielding a parallel-plate capacitor with an initial capacitance of C_0_, expressed as

C_0_ = ε_0_ ε_r_ Ac/d (1)

where A_c_ denotes the overlapped area of electrodes, d is the thickness of the dielectric layer, ε_r_ represents the dielectric constant of the dielectric material, and ε_0_ is the permittivity of vacuum. The capacitance of strain sensors depends on the resistance value of the electrodes, which increases under the stretching due to the changes in the capacitive area [66,150].

**Figure 4:**
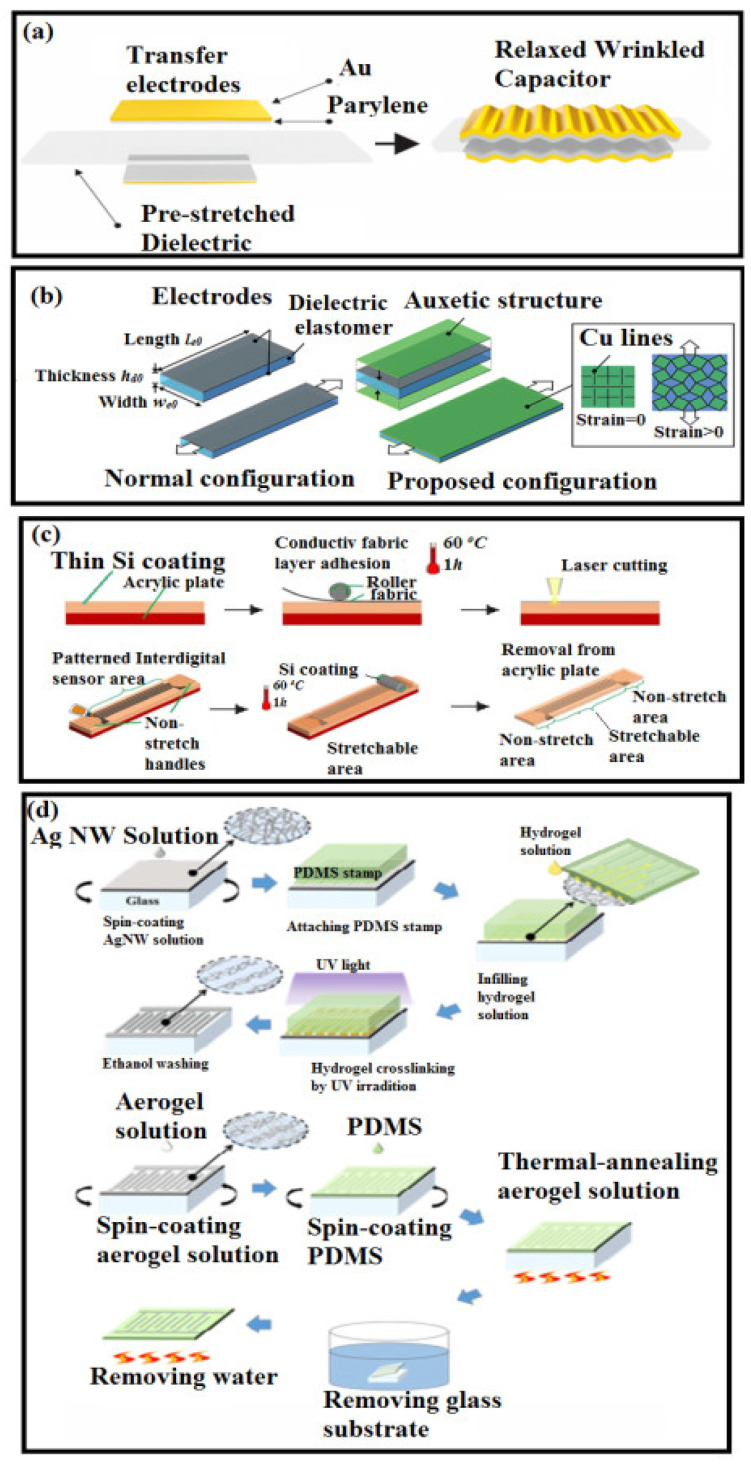
(**a**) Ultrathin wrinkled Au film strain sensor assembly and the structure of the Au film strain sensor [151]; (**b**) capacitive-type hierarchical auxetic structure [152]; (**c**) manufacturing process of the sensor [154]; (**d**) fabrication process of patterned Ag NWs by CFL process and its embedment in PDMS [153].

Resistive-type strain sensors offer high sensitivities and outstanding gauge factors, but a practical application requires characteristics such as linear strain response, high stretchability, and low hysteresis, which are the major limitations of resistive-type sensors, which can be overcome by parallel-plate capacitor structures. Nur et al. fabricated a wrinkled-shape PDMS dielectric layer, thermally evaporated gold on perylene electrodes, and further transferred it onto the PDMS to construct a capacitive type of strain sensor, which is presented in Figure 4a [151]. A layer-by-layer approach is used to double the sensitivity and lower the hysteresis, as presented in Figure 4b [152]. Atalay et al. fabricated a capacitive-type strain sensor (see Figure 4c) with the initial coating of an Eco-flex film on the acrylic plate, and a stretchable, conductive-knit fabric adheres to the silicone. This interdigitated shape is engraved using a laser, and the interdigitated shape is carved out by burning the conductive fabric. Excess fabric parts around the interdigitated shape are removed by peeling them away. Thereafter, the non-stretchable, woven fabric is added as handles, and the silicone elastomer is cast into the structure again. At this stage, a silicone elastomer fills the areas between the electrodes, forming dielectric layers, and it encapsulates the structure. Kim et al. reported a transparent and stretchable thin-film capacitive-type strain sensor, which is presented in Figure 4d, in which a Ag NWs were patterned using the capillary force lithography (CFL) method and were embedded onto the surface of the PDMS substrate [153].

### 3.3. Optical Strain Sensors (OSS)

**Figure 5:**
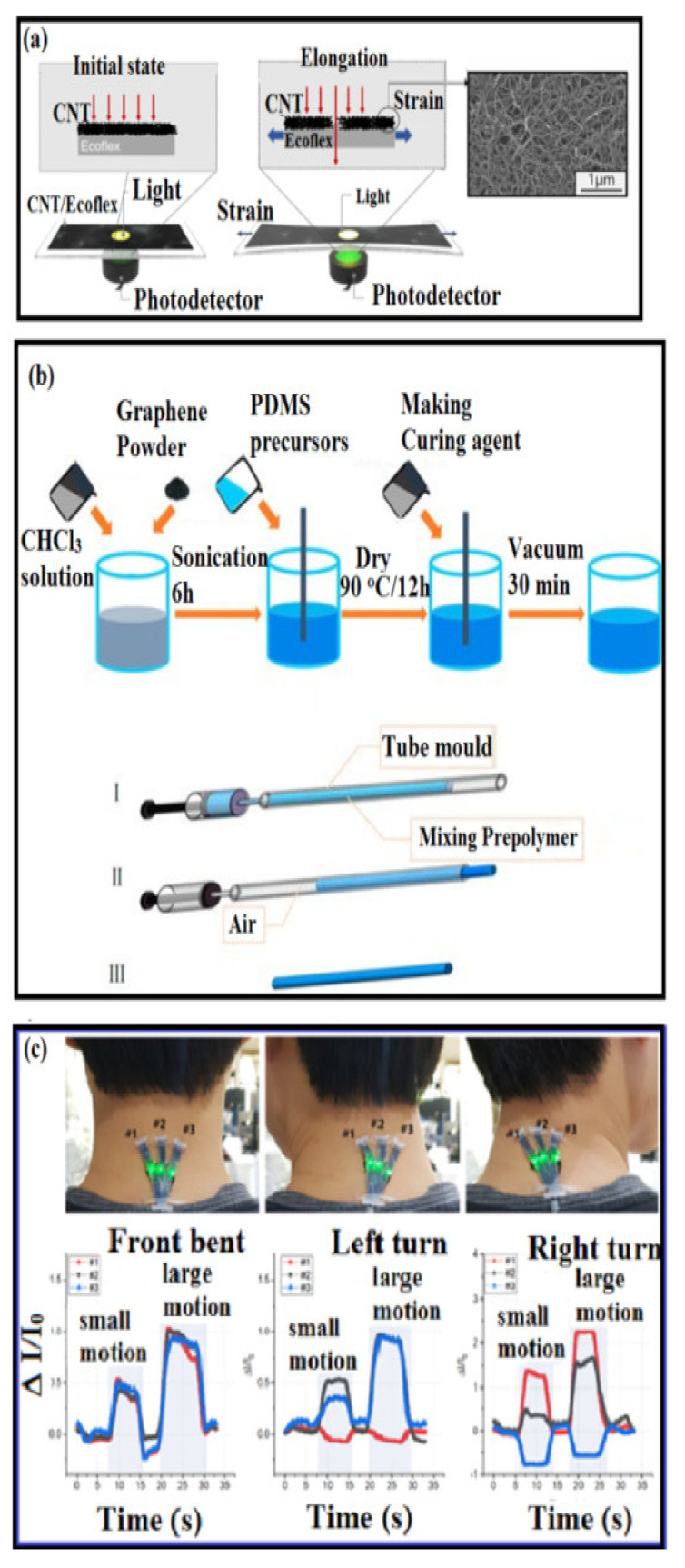
(**a**) CNT-embedded Eco-flex thin-film-based strain sensor based on the optical transmittance change because of the microcrack opening of the CNT network and the scanning electron microscopy image of spray-coated CNTs [156]; (**b**) preparation of the mixture prepolymer and the process for PDMS fiber preparation—see the text for the details [156]; (**c**) the real-time monitoring of the neck posture that can recognize the neck extension (i.e., front bending), left turn, and right turn with a triaxial strain sensor array.

Wearable sensors based on the optical strain principle typically comprise a stretchable waveguide flanked by light emitters and photodetectors. Since the time of the first fabrication techniques in electronics, such as soft lithography and 3D printing, flexible polymeric waveguides have been investigated for wearable strain-sensing applications [68,69,155]. The principal sensing mechanisms behind the OSS are the difference between the incident (initial illumination applied) and the reflected light (light received at the photodetector) upon deformation [68]. The optical types of strain sensors are more promising to overcome challenges (less effective in environmental disturbances) faced during the other two resistive-type and capacitive-type sensors. An optical-type stretchable strain sensor based on the change in optical transmittance of the CNT-embedded Eco-flex film is presented [156]. MWCNTs were spray-coated and embedded into the Eco-flex substrate, and the microcrack propagation in this MWCNT film led to optical transmittance change (Figure 5a–c). The sensor responses were observed to be independent of the intensity of the light source and the strain rate. The sensor was utilized to detect the bending of the finger and wrist for the control of the robot arm. Furthermore, the applications of this sensor to the real-time monitoring of neck posture, carotid pulse, and facial expression were demonstrated [156]. Stretchable OSS have been fabricated from optically transparent polymeric materials such as hydrogels and elastomers [68,69,119,156,157,158]. A graphene/PDMS fiber with high tensile and good transmittance has been demonstrated to detect tensile strain up to 150% (see Figure 5a). Figure 5b shows, the extraction of the prepared PDMS mixture and injected into a silicone tube mold, cured in the natural state for 24 h; (II) the extrusion of the PDMS fiber by air pressure; (III) an optical fiber schematic of the PDMS-fiber [156]. Figure 5c represents the real-time monitoring of the neck posture that can recognize the neck extension (i.e., front bending), left turn, and right turn with a triaxial strain sensor array. The degassed PDMS precursor was injected into a silicone tube mold; once the liquid components were mixed, the mixture was cured to a flexible elastomer. After curing, the fiber was drawn out of the mold using air pressure [158]. This type of optical sensor can easily detect a large range of human joint movement capabilities, including finger bending, wrist bending, elbow bending, and knee bending. Although there have been considerable efforts made to bring the optical-principle-based wearable sensors into real time applications, their lower resolution and poor dynamic performance are still challenges that need to be addressed.

### 3.4. Thermoelectric Strain Sensors

**Figure 6:**
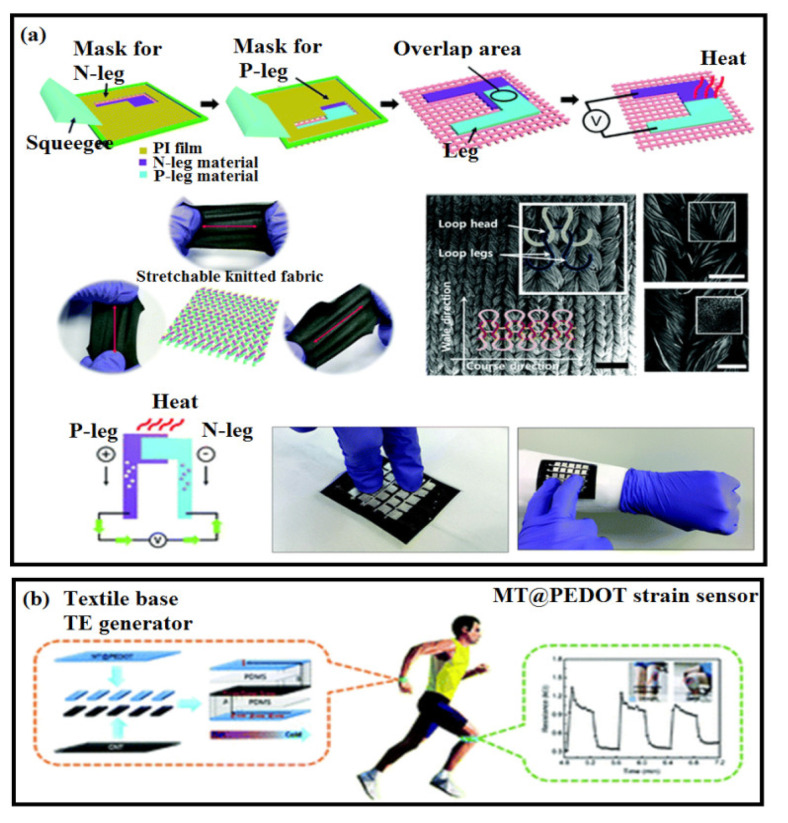
(**a**) Fabrication process for the stencil-printing method, structural characteristics of the stretchable substrate, and material analysis of thermoelectric inks. The SEM images of a pristine knitted fabric illustrated with the knit structure configuration of a loop head and two loop legs (left). Additional SEM images show an enlarged surface with the composite of PEDOT: PSS doped with DMSO (top right) and silver nanoparticles (AgNPs) (bottom right) on knitted fabric. Scale bars are 200 μm. Thermoelectric properties and output characteristics of the temperature sensor and principle of the thermoelectric temperature sensor [159]. (**b**) Assembling a MT@PEDOT-based TE generator device [160].

In the case of TE technology, conventional systems have been considered as a power source for integrated sensors of pressure, corrosion, heat flow, vibration, heart rate, and other stimuli. The utilization of conjugated polymers as the active component/sensitive layer of a TE device is a relatively recent concept, but it has the potential to enable a flexible wearable sensor that can operate at low power and could be fabricated at a low cost. Other desirable attributes, such as low toxicity, ubiquity, and abundance of constitutive elements, as well as the ease in processing by various established coating or printing techniques, are added advantages of thermoelectric strain sensors [46,47]. A highly stretchable and wearable self-powered temperature sensor was fabricated using TE inks such as Ag NPs, graphene, and PEDOT: PSS [159] (see Figure 6a). Firstly, the N-leg (n-type TE material) was printed onto the textile substrate, and then the P-leg (p-type TE material) was printed with an overlapped area of 4 mm^2^, using polyimide (PI) patterned masks for both the N- and P-legs. Images of the multi-axis stretchable knitted fabric are presented in Figure 6a. Among the inks used, PEDOT: PSS/Ag nanoparticle inks were able to generate a high durability of up to 800 cycles of 20% strain. These sensors exhibit temperature sensing properties that are dependent upon the stretching directions, which facilitate applications in human–machine interfaces, health-monitoring technologies, and humanoid robotics.

In another study, a highly conductive PEDOT-based wearable TE strain sensor exhibited an excellent mechanical elasticity and electrical properties in response to external strain. In addition, these sensors showed superior water durability due to the robust PEDOT coatings on the textiles through the in-situ polymerization process. An optimized gauge factor (GF) of the strain sensor reached 54 at a strain of 1.5%, which fully satisfies the demands of wearable electronic sensor devices [160] (see Figure 6b).

### 3.5. Piezoelectric-Based Strain Sensors

Piezoelectricity is a mechanism in which electrical voltage is directly generated under external deformation due to the electrical dipole moments in PE materials (PEM) [161]. Strain sensors based on sensitive materials with a high PE coefficient can induce electric current in an external circuit, leading to the detection of mechanical deformations with high sensitivity and fast response. Therefore, PE materials can usually be used for strain sensors and as energy-harvesters. An active, self-driven wearable PE sensor can be used to monitor respiration rates and rhythm at multiple body peripherals during various physical movements. It is anticipated that self-powered energy harvesters would be highly desirable for next-generation wireless and wearable electronics. The most widely used PE materials, such as lead zirconate titanate (PZT) and lead magnesium niobate-lead titanate (PMN-PT), possess the qualities of hardness, stiffness, and brittle ceramics. However, these PE ceramics are too brittle to integrate with flexible electronics. and hence ferroelectric polymers such as poly(vinylidene fluoride) (PVDF) and its copolymer poly(vinylidenefluoride-co-trifluoroethylene) (PVDF-TrFE) are composited with PE materials as these polymers provide sufficient mechanical flexibility and small polarization, while PE materials offer a sufficient PE response. Hence, excellent and flexible PE materials made from composites [162,163], thin PE films [164], and NFs [165,166,167,168,169] have been intensively investigated for their improved PE performance, cost-effectiveness, and mechanical flexibility [162,163].

**Figure 7:**
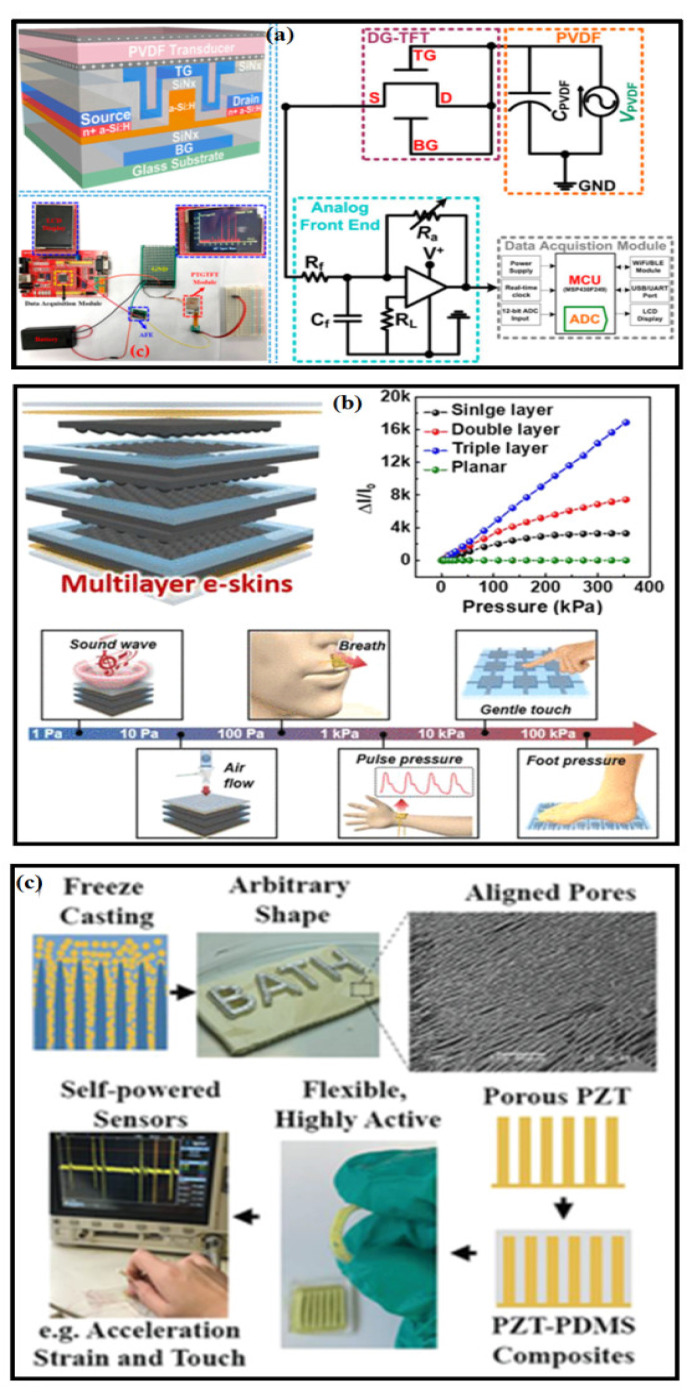
(**a**) Schematic representation for a piezoelectric transistor with its equivalent circuit and the experimental measurements for respiration signal [170]. (**b**) Multilayer piezoelectric e-skin that monitors diverse stimuli in response to foot and wrist pulse measurements [163]; (**c**) a self-powered piezoelectric and pyroelectric multifunctional sensors fabricated using freeze casting [172].

Figure 7 represents the fabrication process for a PE-based strain sensor containing a PE transducer (PVDF) and sandwiched between active sensor materials. Ahmed et al. reported a structure with a PE transducer and a dual-gate thin-film transistor (DG-TFT) to form a PE transducer-gated TFT (PTGTFT), which is shown in Figure 7a. These PE-based sensors have a superior capability of detecting the dynamic force signals and can simultaneously rectify and amplify it [170]. The equivalent circuit diagram for a respiration monitoring system based on PTGTFT is presented in Figure 7a, wherein the shortening bottom-gate (BG), top-gate (TG), and drain (D) terminals of the PTGTFT make a diode-mode connection for signal rectification. In this study, no external power source is used, and all the biasing voltage originated from the self-powered system with ultimately zero power consumption. Park et al. proposed a wearable e-skin using PVDF/rGO micro-dome (PE and piezoresistive) structures [171] (see Figure 7b). These structures were able to differentiate static and the dynamic pressure, for example, in artery vessels, acoustic sound detection, and the surface texture recognition of various surfaces. The artificial fingertip or microdome structures had low sensitivity range of 35 µA Pa^−1^ (2.45 kPa) and 5 µA Pa−1 (2.45–17.15 kPa) and were able to detect skin temperature with a sensitivity of 3.3% per °C. Furthermore, e-skin with three stacked interlocked microdome layers were also fabricated and demonstrated [163]. Figure 7c shows the freeze-casting fabrication process to make a lamellar-shaped PZT-PDMS, where PDMS is impregnated with matrix, and thereby a composite mixture provides the advantages of flexibility from polymer matrix and piezoelectricity from the connected PZT fillers [172]. With different configurations, the self-powered sensors can detect longitudinal, transverse, and shear loads. In addition, with a single PE sensor, it is possible to detect the various mechanical stimuli over a broad sensing range.

### 3.6. Triboelectric Strain Sensors

As mentioned in the past sections, a triboelectric sensor also converts mechanical energy into an electrical response [173,174,175]. When two thin materials with opposite tribo-polarity come into contact with each other, a charge transfer at their interface results in the creation of an output potential [161]. The intensity of the generated potential is directly proportional to the interaction with the external load/deformation regarding time and area. Despite some advantages of PE and triboelectric strain sensors, they usually operate under fast mechanical deformations, and it is difficult to measure the strain history over large stretching cycles because of the fast charge transfer [161,173,174,175].

**Figure 8:**
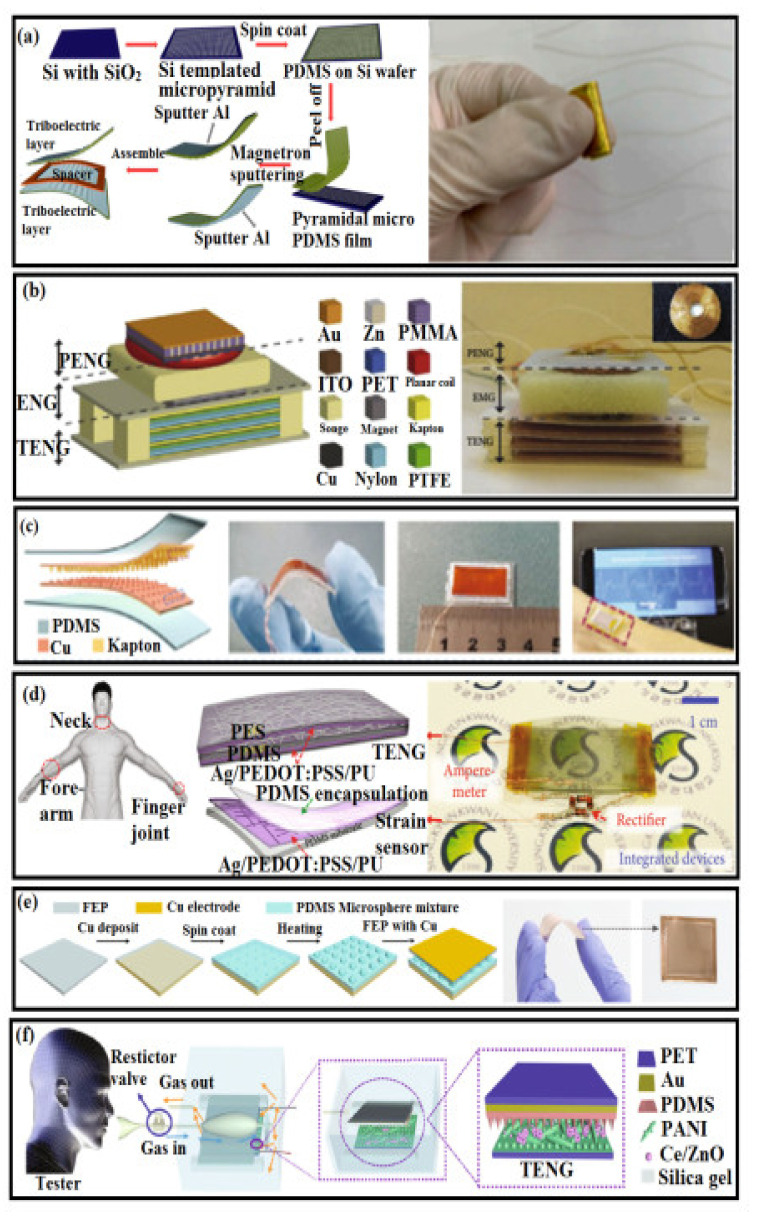
(**a**) Fabrication process of the triboelectric nanogenerator (TENG)-based strain sensor and its flexibility [176]; (**b**) the hybrid nanogenerator is made using a piezoelectric nanogenerator (PENG) and a triboelectric nanogenerator (TENG) [177]; (**c**) the bendable self-powered pulse sensor (SUPS) is made from Cu-Kapton and can be placed over the radial artery [177]; (**d**) patchable integrated devices are made from PDMS-AgNWs/PEDOT:PSS/PU and tied on the neck, forearm, and finger joint [177]; (**e**) strain sensor fabricated from FEP-PDMS with a size of 33 × 33 mm^2^ [177]; (**f**) the human-respiration-driven system was prepared by PDMS-PANI [177].

Microstructured PDMS films/PET spacer layers were constructed using a triboelectric nanogenerator that could fully contact human skin and be used for harvesting mechanical energy and detecting human motion. Initially, a 200 nm Al was sputtered on the front of the micro-pyramid-structure PDMS film as both the friction layer and the electrode, and the 200 nm Al was sputtered on the back of another micro-pyramid-structure PDMS film as another electrode. Then, a spacer layer (PET) was placed between the two PDMS films. Further, the conductive fabric was placed on the Al film to form an external contact. Finally, the device was covered by the pure PDMS film as a protective coating. Due to its wearable and flexible nature, the well-designed triboelectric generator provides a new way to achieve a power supply for wearable electronic devices and act as an active sensor to monitor human gesture movements (see Figure 8a) [176]. The energy generated by human motion was collected by multi-layered electrodes in triboelectric nanogenerators (TENGs). An Al foil was used as both electrodes and as a friction layer, a fluorinated ethylene propylene (FEP) film served as the friction layer, and copper deposited on it served as the second electrode. The TENG in shoe insoles could be driven to generate a voltage of about 700 V output and a short circuit transfer charge of 2.2 mC. This provided a new kind of TENG that could work in different conditions. Ouyang et al. fabricated a self-powered pulse sensor (SUPS) based on a triboelectric active sensor [119]. The SUPS was composed of four parts: friction layers, electrodes, a spacer, and encapsulation layer. Nanostructured Kapton (n-Kapton) film served as one triboelectric layer, and the Cu layer deposited on its backside acted as one electrode. The nanostructured Cu (n-Cu) film served as both a triboelectric layer and an electrode (Figure 8b–f). The fabricated SUPS was ultrasensitive and low in cost, which is important for any wearable sensors [176].

### 3.7. Field-Effect Type Strain Sensors

To utilize the stretchability of a polymer or plastic, metal electrodes were used on rubber substrates. The embedded active components, such as transistors and diodes in rubber sheets, are integrated with wavy metal wires [178]. As an essential element of various intelligent electronic devices such as organic thin-film transistors, they possess the integrated functionality of signal transduction and amplification. In principle, the combination of the above properties is ideal for e-skin and health monitoring sensing applications [45,58,59,179].

**Figure 9:**
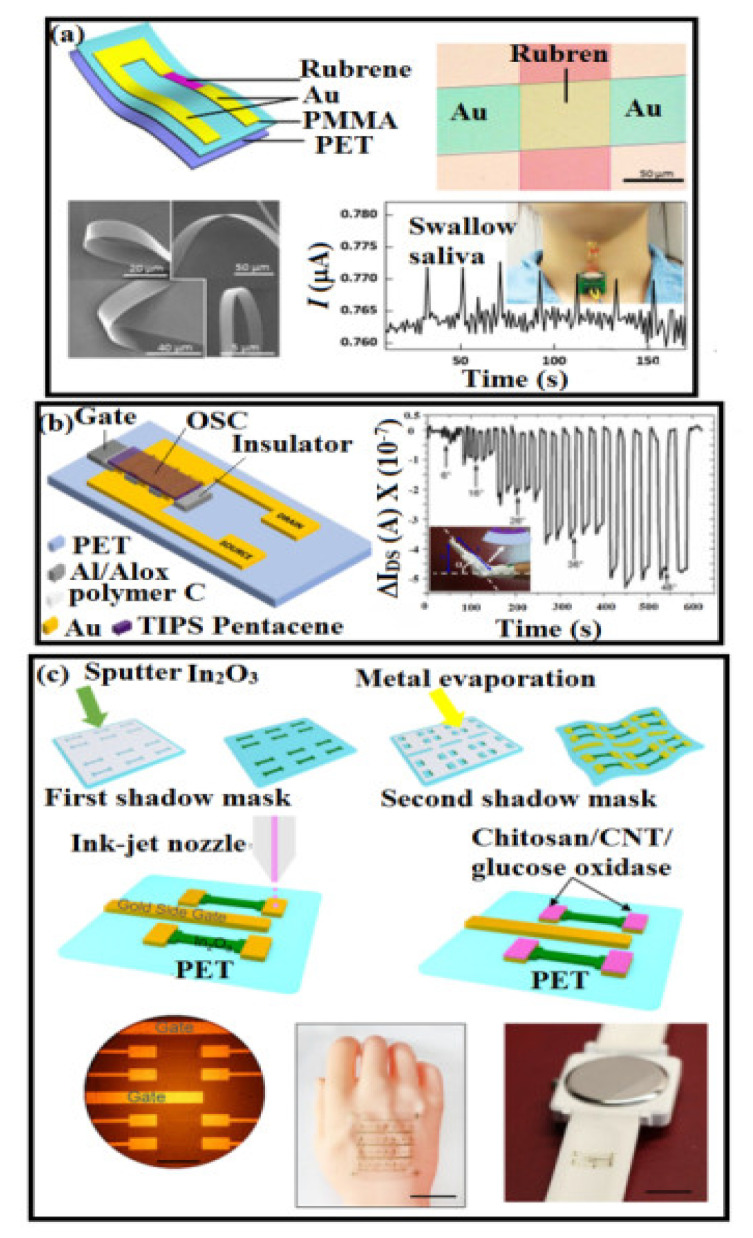
(**a**) Schematic illustration of flexible sensors based on a flexible rubrene single crystal showing an optical microscopy image of the real device, the flexibility confirmed by SEM and showing its flexibility in an SEM image [184]; (**b**) fabrication process for the FET based strain sensors and its application to wrist motion detection [185]; (**c**) optical image showing In_2_O_3_-based biosensors with two gold side-gate electrodes (scale bar is 500 μm). A photograph of In_2_O_3_ FET foil laminated on an artificial human hand. The scale bar is 3 cm [64].

Various strain sensors have been reported to date; among them, organic strain sensors are promising due to their low cost, good flexibility, light weight, and easy fabrication process. In general, these sensors are based on an organic-semiconductor and three-terminal OFET configuration [180,181,182]. Sekitani et al. reported that pentacene thin-film OFET demonstrated compress strain, which led to an increase in field-effect mobility [183]. The inverter-type thin-film transistor circuit was also reported to improve the gauge factor of up to 1.51 and successfully achieved the detection of the finger motion [180,181,182]. As shown in Figure 9a, rubrene single-crystal-based sensors were made to detect swallowing saliva movement [184]. A polymer-based FET strain sensor was used to measure wrist motion, which is presented in Figure 9b [185]. The fabrication process of In_2_O_3_ FETs on a PET substrate using two-step shadow masks followed by a schematic diagram for an electrode ink-jet printed electrode are illustrated in Figure 9c [64].

## 4. Sensing Mechanisms

Sensing mechanisms depend on the type of sensor (for instance, the capacitive type and the optical type), the type of sensing materials, their surface interaction with supporting materials, and the fabrication process. For instance, the resistance of resistive-type sensors can be modified with respect to their geometry (length, cross-section) upon external stretching. The geometric effect is more dominant in liquid–metal-based stretchable strain sensors [186,187,188]. For instance, elastic hollow fibers composed of triblock copolymer resin were fabricated into strain sensors, and a liquid metal alloy (eutectic gallium indium) was injected into the cavity for better conductivity [189]. In another study, PDMS fibers were injected with low toxicity liquid metal that enabled them to detect strain variation in geometrical changes [190].

Geometric effects play an important role in capacitive and optical strain sensors, changes in the capacitive area, and the thickness of the dielectric layer, leading to a shift in the capacitance as a function of either external stretching or compression. Whereas the optical type of strain sensors relies on the attenuation in the light transmission on the stretching of optical waveguide [68,119,191], the majority of sensing mechanisms are based on the intrinsic resistive response of the sensing materials. This could be defined by the change in the electrical resistance of the materials in response to external deformations. The intrinsic resistance of a sensor dramatically increases upon changes in the bandgap, and carbon nanotubes and oxide-based nanowires endure a very high resistive response, which leads to the development of highly sensitive strain sensors incorporated into highly stretchable substrates. However, large mechanical mismatch and weak interfacial adhesion between micro-/nanoscale materials and stretchable supporting materials dramatically lower the contribution of their intrinsic resistive response to the overall sensing performance of stretchable strain sensors. The sensing behavior of resistive-type strain sensors can be attributed to the changes in the conduction network upon deformations. As per the percolation behavior, a minimal number of nanomaterials is required to establish conducting pathways within a film or composite. Once the adjacent nanomaterials are connected, electrons can pass through established conducting networks. Upon stretching, nanomaterials-based resistive-type strain sensors lose their overlapping area and electrical connection, which leads to increased overall electrical resistance. The disconnection/mismatch is because of large stiffness between nanomaterials and stretchable supporting materials, leading to slippage and debonding of nanomaterials in the context of major stretching. Nanowires and nanoflakes take greater advantage of the disconnection mechanism. The resistance shift in CNT-based thin films has been reported to be due to changes in interconnecting pathways during the stretching and destretching [84]. A conductive network of Au nanosheets has also produced similar conductive networks [192]. Upon stretching, the nanosheets slip toward the direction of the external load, decreasing the contact area between them and thus increasing the overall resistance of the strain sensor. In addition to disconnections, cracks also appear on top surfaces of the soft polymers or natural fibers upon stretching. Cracks propagate in stress-concentrated areas. Although the cracks are undesirable, microcracks have been utilized in conductive thin films to develop highly sensitive strain sensors. Microcracks have been observed in CNT-based sensors [102,193], graphene-based strain sensors [98,194,195] and graphene derivatives [92,196], metal nanowires, and nanoparticle-based [197,198] strain sensors. The rapid separation of nanomaterials at the microcrack edges dramatically limits the electrical conduction paths within the thin films, leading to a significant increase in the electrical resistance of strain sensors under the applied tensile strain [66]. Recently, controlled cracks have been utilized as an effective mechanism to promote the sensitivity of strain sensors [113,198]. Au thin-film-based strain sensors have been fabricated on PDMS substrate to study the effect of cracks on the sensitivity and electrochemical response [194]. In one study, graphene-based ultra-sensitive and stretchable strain sensors fabricated with reversible parallel microcracks [199]. The length and density of microcracks were increased with applied strain, and microcrack edges were reconnected upon releasing the strain, ensuring the recovery of the electrical resistance after the complete strain release. Strain sensors are based on natural fibers, and the fiber breakage in the direction of the applied strain leads to crack propagation and subsequent increase in electrical resistance [116]. Natural-fiber-based strain sensors with high sensitivity and stretchability and with controlled crack propagation and fiber breakage have been fabricated [117,119]. Electron tunneling occurs when electrons pass through a gap between two conductive nanomaterials with a short distance between them [66]. Conductive nanocomposites made of functional nanomaterials and polymer matrices have not only direct electrical paths through connected nanomaterials but also tunneling conduction between adjacent nanomaterials [65,67]. The minimum distance (nonconductive barrier) through which electrons pass through is to create a quantum tunneling junction is called the cut-off distance. The cut-off distance depends on several factors, including the type of insulating material, conductive fillers, and processing parameters. The tunneling resistance originated from quantum electron junctions. In CNT-based nanocomposite strain sensors, the strain response arises from changes in the tunneling resistance [200,201]. In nanocomposite strain sensors, CNTs are often entangled and self-folded within polymeric matrices. When stretched, entangled CNTs are more susceptible to unfolding rather than sliding, leading to changes in the tunneling resistance among neighboring CNTs. It is important to point out that the tunneling effect differs from the disconnection mechanism where many connected networks are separated due to the slippage of nanomaterials within polymer matrices.

In addition to the affecting factors, the gauge factor is a quantifier for a sensitivity of a strain sensor, which is defined by the ratio between the relative change in the output signal to the applied strain [65]. The value of a gauge factor of a strain sensor depends on various factors such as sensing microstructures, elements, fabrication process, and sensing mechanism [138,202]. For example, the gauge factor of a resistive-type strain sensor is defined as a gauge factor = *(*Δ*R/R*_0_)*/ε*, where Δ*R/R*_0_ is the relative change of resistance and *ε* is the applied strain. Zhou et al. fabricated a CNT-PDMS-composite-based resistive type strain sensors showed sensitivity 10^7^ at 50% strain [203]. High sensitivity was attributed to the carbon nanotube networks in the cracked area. Highly stretchable and ultrasensitive (gauge factor of 88,443 at 350% strain) strain sensors made of 3D printed graphene and PDMS nanocomposite followed by plasma treatment, and polyethyleneimine coating, and finally, the deposition and reduction of GO particles was reported [204]. The gauge factor originates from the disconnection of the rGO at low strain range, whereas the open mesh structure can enhance the sensitivity at high strain levels. Different studies have shown a wide range of gauge factor values starting from 1.2 to 102,351 [111,113,116,117]. It has been noticed that the gauge factor of the strain sensors can be controlled by geometric engineering [111]. Serpentine-shaped active materials exhibit lower sensitivity than straight ones, and a strain sensor based on the fragmented conductive cotton fabric-Eco-flex composite showed ultra-high sensitivity up to 102,351 within the strain range of 342–400% [113].

The maximum theoretically achievable gauge factor value for capacitive-type strain sensors is about one (gauge factor = (Δ*C*)/*C*_0_*ε*  =  ((1  +  *ε*) *C*_0 _− *C*_0_)/*C*_0_*ε*  =  1), which means that most of the stretchable capacitive-type sensors have gauge factor values less than one (gauge factor 1). Carbon-black-filled elastomer composite and silicone elastomer-based strain sensors showed gauge factor value of about 0.98 at 500% stretchability [113]. Capacitive-type stretchable sensors with hollow elastomeric fibers filled with liquid metal networks reported gauge values of up to 0.82 [205]. Nevertheless, this theoretical limitation of capacitive-type sensors has demonstrated the possible enhancement of sensitivity by more than one (gauge factor 1) due to geometrical alterations and novel material formulations [151,206]. A capacitive-type strain sensor using Au-film electrodes achieved a gauge factor of about 3 under 140% strain [206]. In another study, highly sensitive capacitive-type sensors have been reported using ionic hydrogels, and Ag-nanofiber-based nanocomposites have achieved a very high gauge factor of 165 under an ultrahigh strain of 1000% [151]. The improvement in sensitivity is because of the incorporation of Ag nanofibers leading the hydrogel–metal interface. In optical-type strain sensors, the sensitivity is quantified by an output power loss of a stretchable waveguides under mechanical deformations [68,207]. In a study, the sensitivities of stretchable strain sensors made of thermoplastic elastomer and dye-doped PDMS optical fibers were 10 and 3.62 dB ε^−1^, respectively [69,119]. CNTs-embedded Eco-flex film-based optical-type stretchable strain sensors achieved high sensitivity with gauge factor ≈ 30 [208].

## 5. Real-Time Healthcare Monitoring

**Figure 10:**
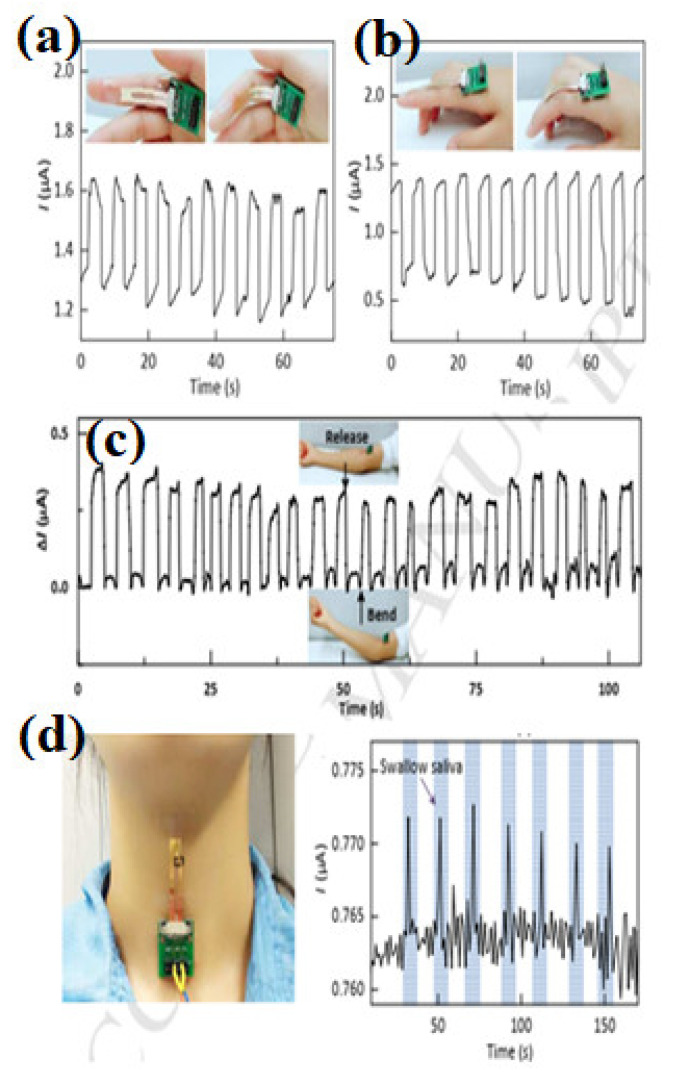
Real-time current response of rubrene single-crystal strain sensor under the motion of the index finger corresponding to (**a**) applied compressive strain and (**b**) tensile strains. (**c**) Real-time current response of the device with arm motion before and after bending. The insets are the photographs of the device under bending and releasing; (**d**) the real-time current response of the strain sensor attached to the throat to detect swallowing saliva movement [184].

**Figure 11:**
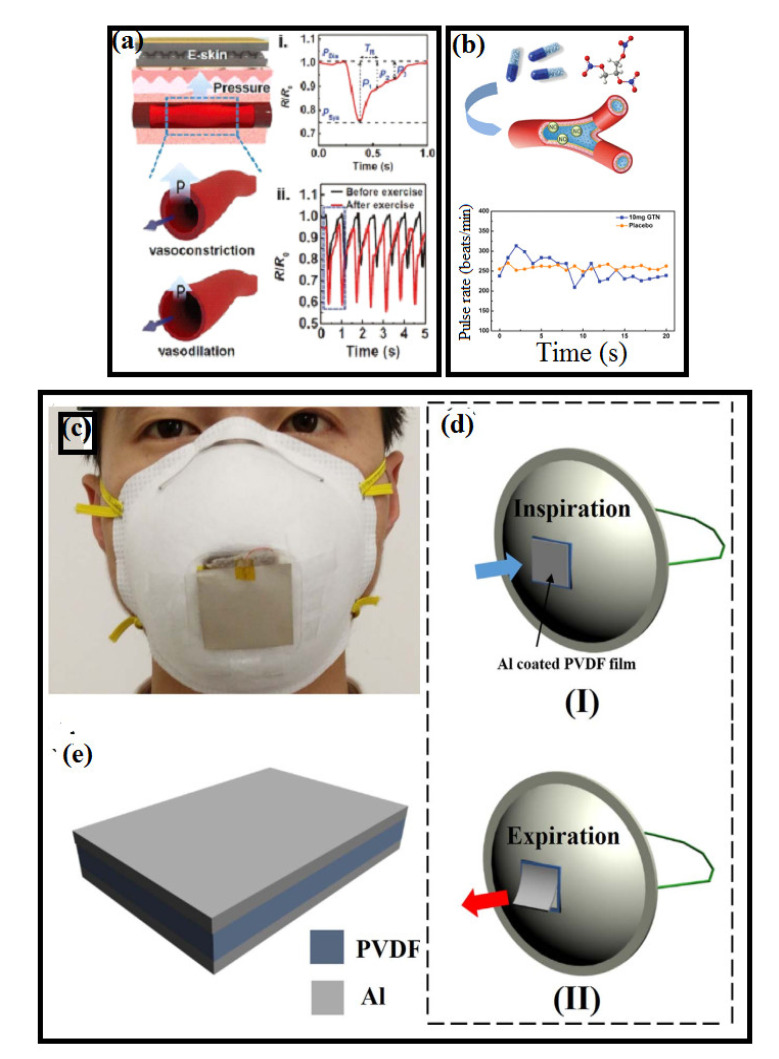
Perception of cardiovascular and respiratory signals with wearable and skin-attachable pressure/strain sensors; (**a**) fingertip-skin-inspired ferroelectric skins for the detection of (i) artery pulse pressure and (ii) variations in pulse waveforms before and after exercise [171]. (**b**) Monitoring the effect on the cardiovascular system of rabbits. (**a**) Diagram of the process of the nitroglycerin effect on the cardiovascular system of rabbits and changes in the heart rate of rabbits within 20 min of taking the placebo and glyceryl trinitrate (GTN) [213]; (**c**) the physical photo of the wearable pyroelectric nanogenerator (PyNG); (**d**) schematic of a wearable PyNG driven by human respiration—(I) Inspiration, (II) Expiration; (**e**) schematic of the pyroelectric PVDF film [217].

SSSs have been used in different applications such as the monitoring of human body movements, respiration, heartbeat, and body joints by interfacing human–machine correspondence with the aid of robotics, which has marked significance in the context of intelligent sensors [209]. As shown in Figure 10, a rubrene single-crystal device that is highly sensitive, flexible, and stable could be potentially used in the real-time monitoring of large scale motions [184]. From Figure 10a, the strain sensor is attached to an index finger in order to capture its motion, which is measured by the change in current with the application of a constant voltage of 10 V. The current either increases or decreases, as shown in Figure 10a,b, which is consistent with the inside-bending and outside-bending of the index finger. These rubrene single crystals have also been used to monitor the arm motion detection (see Figure 10c); after 24 cycles of arm bending, the current change returns to its initial value. As presented in Figure 10d, the strain sensor is attached to the throat in order to capture the swallowing of saliva by detecting the motion of the Adam’s apple or laryngeal prominence. For respiration monitoring, a strain sensor was mounted onto the chest area [210] to detect the cardiac impulses or apexcardiogram (ACG), which are respiration signals that could potentially be used for diagnosing the ventricular abnormalities and heart diseases for several decades [211]. SSSs have also been utilized for the detection of emotional expression and phonetic recognition [193,210]. Shi et al. reported the use of combined properties of AgNWs/graphene-nanoplate-based strain sensors attached onto the human body to detect various human motion signals, including wrist, radial, and ulnar arteries [44,212]. In order to non-invasively detect cardiovascular and respiratory signals (see Figure 11a), ferroelectric wearable sensors were attached to the wrist and accurately measured systolic, diastolic, and reflected pulse waves [171]. Polymer-based wearable sensors have also been used to detect pulse waves, such as the heartbeat, and blood pressure [213]. These sensors work based on the capacitance change induced by the hole carriers in the semiconducting polymer (see Figure 11b), and a glyceryl trinitrate (GTN)-based transducer was attached on the chest of rabbits (male New Zealand White rabbits of 2.4–2.6 kg). The effect of GTN on the cardiovascular system can be detected by using a transducer [213]. It was also demonstrated that the graphene/CNT composite framework can detect the pulse waveforms of jugular venous stimuli on the neck as well as breathing in pregnant and healthy subjects [214]. Due to the high sensitivity and the low detection limit of Ti_3_C_2_Tx-based strain sensors, it could be used to monitor the subtle motions of the human body [215]. To measure the heartbeat of an athlete, a strain sensor was attached to the chest, and the response of the breath was captured as small peaks corresponding to the heartbeats [216]. Xue et al. reported wearable breathing pyroelectric PVDF sensors that were located on the respirator at the location where the airflow of the breath is the most concentrated (Figure 11c) [217]. As shown in Figure 11e, a wearable pyroelectric nanogenerator was driven by human respiration—(I) inspiration and (II) expiration—and the schematic is given for the pyroelectric PVDF film [217]. To monitor the phonetic movements, a strain sensor was attached at the throat of the test person [193,197].

Furthermore, the strain sensors were attached to the vocal cords of the healthy individuals at the time of pronouncing the words/phrases “hello”, “graphene”, “sensor”, and “fish scale”. The strain sensors produced the repeatable signals that were distinguishable, and each word has a unique amplitude and duration [218]. This phonation recognition enables this type of sensor to be used in potential applications in human/machine interaction and phonation rehabilitation [37,110]. In addition, Xu et al. demonstrated a real-time motion monitoring at different bending angles of the elbow [219]. The stretchable strain sensor was also able to detect fast elbow movement, which required high signal reproducibility of the device [219]. The strain sensors were also attached within the glove wired from each finger to determine the different fingers for the change in voltage [199]. Despite the direct attachment of the sensor onto the human body, Ouyang et al. reported a kinesthetic sensing glove for ambulation in order to evaluate the residual hand functionality and recovery condition of the patient post-stroke [219]. It was noticed that the change in resistance was directly dependent on the bending angle and the motion level.

These results suggest that the stretchable strain sensor can successfully detect pathological movements without electromyogram (EMG) electrodes. However, it is understood that the strain signal experienced through direct attachment was larger than the glove attached. Further, it was extended to measure large joint movements [109]. In recent years, capacitance-based sensors have also been explored in monitoring human psychological changes, for example, monitoring diseases such as Alzheimer’s [207]. The sensor was mounted around the mouth and smiling and wrinkling were recorded. The smiling action (mouth muscle) induced strain on the sensor, causing a decrease in capacitance, but there was no prominent signal from not smiling. Similarly, the sensor was attached to the corner of the eye, and the sensor could detect the movements between eye-closing and eye-opening scenarios. When an eye opens, the muscle deforms with much larger strains, which results in a reduction in the capacitance. Ozgur Atalay et al. demonstrated the use of a capacitive-based strain sensor in wearable devices; this strain sensor was used to monitor knee joint movements [12]. To monitor knee movement, the soft sensor was mounted onto an elastic, knitted, and tight fabric using Velcro, which was worn by the subject [12]. The capacitance values of the soft strain sensor changed as the knee joint moved with different strengths and frequencies. Moderate bending of the knee produced a larger change in the capacitance value compared with slight bending, which is because the moderate bending stretches the soft sensor structure more. The soft sensor also successfully recorded walking and running actions. Moreover, the frequencies of walking action and running action were calculated as 0.73 and 1.61, respectively. It is very hard to monitor complicated human body deformation due to the multidirectional movement, which cannot be accurately detected by the individual strain sensor. To monitor such strains in various axes, it is important to integrate several strain sensors into multi-axial strain sensors [110]. Zhang et al. designed a rosette-shaped strain sensor configuration consisting of three graphite/silk fiber (GSF) strain sensors [3]. These sensors were organized at the same end toward the center with an intersecting angle of 120°. The authors mounted this rosette-shaped strain sensor onto the wrist (one strain sensor was parallel to the arm and the other two were located alongside it) and monitored human motions in multiple directions. Textile-based WSSs were also applied to monitor real-time robot movement. Graphene fiber strain sensors across joints were explored to accomplish the real-time detection of movements. In another study, Jin-Woo Park et al. developed interdigitated capacitive strain sensors for sensing body motions [111]. The motions of the finger and wrist muscles were measured in order to simulate large and small strain sensing, respectively. There was a stable and predictable variation in the capacitance of the sensors when the finger and wrist muscles were set into repeated motion, and the folding of the finger resulted in a decrease in the capacitance of approximately 10% [112]. For large movements, strain sensors were mounted on the radial artery of the wrist for blood monitoring. The waveform with a frequency range of 70 beats/min was noticed within the normal range of a healthy person [193,220]. Additionally, strain sensors were fixed on the wrist in order to evaluating sensing activity at different angles and were observed as 5.49, 13.39, and 23.65%. These results suggest repeatability and reliability for the monitoring of large motions [221].

**Figure 12:**
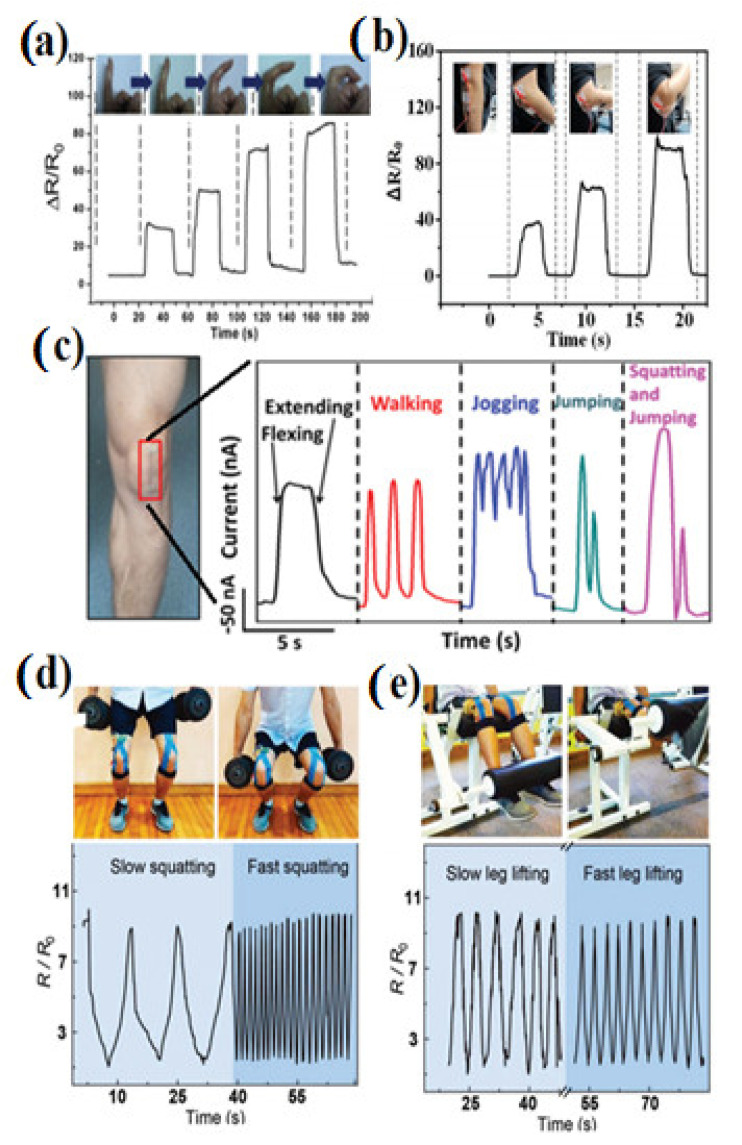
Applications of strain sensors in sport activity. (**a**) The strain response from the resistance strain sensors of finger movements [197]; (**b**) resistance change of a sensor with arm bending at various angles [142]; (**c**) wearable sensor attached to the knee and its responsive signals under knee motions flexing/extending, walking, jogging, jumping, and squatting/jumping [216]; (**d**) a sensor was attached onto the joint of a lower limb to monitor slow and fast squatting actions, and the corresponding resistance change was measured [223]; (**e**) a fiber-shaped sensor was attached to leg, detecting leg lifting as slow and fast motions, and provided corresponding signal [223].

In the context of sports, strain sensors have been also explored in assessing the physical and mechano-chemical parameters of an athlete [216,222]. Soft wearable strain sensor devices interfacing with the body allow new integrated platforms to continuously monitor both biophysical and biochemical signals of an athlete’s performance. In contrast, skin-interfaced wearable devices in stretchable forms can closely couple with various locations on the body, offering highly localized monitoring. Figure 12a shows a stretchable strain sensor fixed at the joints of the index finger and its corresponding output signal during repeated bending and straightening of the finger. The magnitude of the signal increases with an increasing bending level starting from 0 to ~80% [193]. It was also mentioned, as shown in Figure 12b, that the sensor is attached under the arm to monitor and also distinguish different bending angles of the arm [142]. Figure 12c shows a strain sensor embodied with commercial accelerometers to capture knee motions. The responsive curves of a sensor on the knee are also shown in the context of flexing/extending, walking, jogging, jumping, and squatting/jumping motions [116,117]. Biophysical parameters, such as respiration and heart rates, provide deep insights into both the physiological health state of an athlete before, during, and after physical activity, and the efficiency of different training regimes [118]. In the event of squatting, the sensor was attached to the leg and monitored the squatting at slow and fast speeds [223]. It also observed the bending angles. As shown in Figure 12d, the sensor was attached to the joint of the lower limb to monitor slow and fast squatting actions, and the corresponding resistance change was measured. The magnitude of the squatting signal for slow and fast was the same. Figure 12e represents the sensor attachment in the context of leg lifting and its corresponding change in resistance (for the slow and fast movements). On top of the sensor, the fiber-shaped strain sensor can weave into the clothes and monitor the bending and other action of sports wearers [223].

## 6. Limitations and Challenges

Nanomaterials-based WSSs have demonstrated potential applications in human motion detection and the monitoring of positions in various parts of the body. However, the challenges associated with the design, integration, and safety of wearable SSSs still exist, and with the evolving technology in the field of nanomaterials, the challenges can soon be overcome. The measurement of decoupled strains in multiple directions and multi-plane deformations is still challenging, and the use of novel sensing nanomaterials, architectures, 3D structures, and other metamaterials could be a plausible way to overcome these limitations [66,224,225]. One study managed the major challenge of temperature self-compensated hybrid film (graphite/CNTs) to decouple the effect of temperature change on the piezo resistivity of strain sensors [226]. Additionally, other challenges such as preventing water molecules from coming into the sensing area were overcome by using super-hydrophobic coatings [227,228]. The humidity variations can be enhanced with strain sensors by sandwiching mechanisms using Eco-flex–CNTs–Eco-flex methods [229]. Noise-free and accurate strain monitoring is another consideration and the adhesion of WSS onto the skin must be conformal; weak adhesion will lead to delamination from the skin [220]. To overcome this challenge, pressure-sensitive adhesives [230,231], gecko-inspired structures [232,233], swellable microneedle arrays, [234,235], and ultra-thin packaging [236,237] have been pursued, and still new methodologies are being adopted. However, strong adhesion is still a hurdle; a flexible skin-adhesive film composed of PDMS microfibers decorated with vinyl siloxane tips was proposed and used successfully to increase the adhesion strength of up to 18 kPa [220]. The most important challenges are biocompatibility and health safety when using these sensors on the skin for a long time, which can irritate the patient and lead to bacterial infections, creating discomfort [238]. Again, by using breathable wearable devices 3D micro-architectures, fiber-structured films, and porous polymers, this challenge can be overcome [220,239,240]. A nanomesh, ultra-thin, and gas-permeable wearable device was made to suppress skin inflammation, which is another hurdle to be overcome [241]. It is worth mentioning that research should focus more on biocompatible and clinically acceptable materials for wearable strain sensor applications. Although notable advancement in WSS is still challenging because of the technical complexity in the manufacturing process [66,242], it is very important for device integration and digital manufacturing in order to improve functionalities and bring about longer lifetimes [243].

## 7. Conclusions and Prospects

This review summarizes recent technological advances in the development of stretchable devices and WSSs. Different types of mechanisms were discussed separately, and it was envisioned that SSS will expand their impact in applications of human motion detection and healthcare; however, there are several challenges associated with the design of multi-directional, skin conformal, breathable, and integrated WSS that should be further addressed. The enormous success in the fabrication of WSS devices has been made, which is promising for the future of stretchable devices and WSS [244,245,246]. Transparency and touch panels are other key considerations for future wearable applications. At the same time, inorganic metal oxide (ITO, FTO), organic sensing materials (graphene and CNTs) are transparent, active, and self-healing materials that have demonstrated promise for use in future potential applications. A great accomplishment has been achieved in developing enhanced sensitivity, stretchability, flexibility, superior transparency, and easy-to-obtain signal output behaviors that will render wearable and portable sensing devices a fresh and novel area.

## Data Availability

Not applicable.
